# Sclerostin modulates mineralization degree and stiffness profile in the fibrocartilaginous enthesis for mechanical tissue integrity

**DOI:** 10.3389/fcell.2024.1360041

**Published:** 2024-06-04

**Authors:** Shinsei Yambe, Yuki Yoshimoto, Kazutaka Ikeda, Koichiro Maki, Aki Takimoto, Akihide Tokuyama, Shinnosuke Higuchi, Xinyi Yu, Kenta Uchibe, Shigenori Miura, Hitomi Watanabe, Tetsushi Sakuma, Takashi Yamamoto, Kotaro Tanimoto, Gen Kondoh, Masataka Kasahara, Toshihide Mizoguchi, Denitsa Docheva, Taiji Adachi, Chisa Shukunami

**Affiliations:** ^1^ Department of Molecular Biology and Biochemistry, Division of Dental Sciences, Graduate School of Biomedical and Health Sciences, Hiroshima University, Hiroshima, Japan; ^2^ Department of Orthodontics and Craniofacial Developmental Biology, Applied Life Sciences, Graduate School of Biomedical and Health Sciences, Hiroshima University, Hiroshima, Japan; ^3^ Laboratory of Biomechanics, Institute for Life and Medical Sciences, Kyoto University, Kyoto, Japan; ^4^ Department of Pharmacology, Tokyo Dental College, Tokyo, Japan; ^5^ Department of Maxillofacial Anatomy and Neuroscience, Division of Oral Health Sciences, Graduate School of Biomedical and Health Sciences, Hiroshima University, Hiroshima, Japan; ^6^ Laboratory of Integrative Biological Science, Institute for Life and Medical Sciences, Kyoto University, Kyoto, Japan; ^7^ Division of Integrated Sciences for Life, Graduate School of Integrated Sciences for Life, Hiroshima University, Higashi-Hiroshima, Japan; ^8^ Oral Health Science Center, Tokyo Dental College, Tokyo, Japan; ^9^ Department of Musculoskeletal Tissue Regeneration, Orthopaedic Hospital König-Ludwig-Haus, Julius-Maximilians-University Würzburg, Würzburg, Germany

**Keywords:** sclerostin, *Sost*, fibrocartilage, mineralization, fibrochondrocytes

## Abstract

Fibrocartilaginous entheses consist of tendons, unmineralized and mineralized fibrocartilage, and subchondral bone, each exhibiting varying stiffness. Here we examined the functional role of sclerostin, expressed in mature mineralized fibrochondrocytes. Following rapid mineralization of unmineralized fibrocartilage and concurrent replacement of epiphyseal hyaline cartilage by bone, unmineralized fibrocartilage reexpanded after a decline in alkaline phosphatase activity at the mineralization front. Sclerostin was co-expressed with osteocalcin at the base of mineralized fibrocartilage adjacent to subchondral bone. In *Scx*-deficient mice with less mechanical loading due to defects of the Achilles tendon, sclerostin^+^ fibrochondrocyte count significantly decreased in the defective enthesis where chondrocyte maturation was markedly impaired in both fibrocartilage and hyaline cartilage. Loss of the *Sost* gene, encoding sclerostin, elevated mineral density in mineralized zones of fibrocartilaginous entheses. Atomic force microscopy analysis revealed increased fibrocartilage stiffness. These lines of evidence suggest that sclerostin in mature mineralized fibrochondrocytes acts as a modulator for mechanical tissue integrity of fibrocartilaginous entheses.

## 1 Introduction

Cartilage is an avascular, aneural, and alymphatic connective tissue that encompasses hyaline, elastic, and fibrocartilage (Shukunami et al., 2008; Takimoto et al., 2009; Hall, 2015). The most prevalent type is hyaline cartilage, characterized by chondrocytes producing significant amounts of type II collagen (Col2) and aggrecan (Acan). Hyaline cartilage includes the cartilaginous bone primordium, acting as a template for future bone development, and the articular cartilage permanently covering joints to safeguard the epiphyseal bone surface (Hall, 2015). Fibrocartilage, with an intermediate appearance between hyaline cartilage and dense regular connective tissues like tendons and ligaments (Benjamin and Evans, 1990; Hall, 2015), is present in the pubic symphysis that is an immobile joint connecting the pubic bones, inner annulus fibrosus of intervertebral discs in the spine, knee menisci within the knee joint between the femur and the tibia, articular discs of the temporomandibular joint that maintains jaw movement and stability, and fibrocartilaginous entheses between tendons or ligaments and bones. In the musculoskeletal system, fibrocartilage acts as a shock absorber and reinforces weight-bearing areas.

The enthesis serves as the attachment site for functional dense connective tissue components, such as tendons, ligaments, joint capsule, or fascia, into the bone in adults, and also to hyaline cartilage in fetal to childhood (Benjamin et al., 2006). Fibrocartilaginous entheses are situated at the epiphysis or apophysis of the bone, while fibrous entheses directly attach to the diaphysis (Sugimoto et al., 2013a; Apostolakos et al., 2014). Previous studies, including our own, have indicated that primordial entheses arise from Scx^+^/Sox9^+^ progenitors during development, forming a junction between the dense connective tissue component and hyaline cartilage (Sugimoto et al., 2013a; Blitz et al., 2013).

In fibrocartilaginous entheses, comprising tendons, unmineralized and mineralized fibrocartilages, and subchondral bone, the Gli1^+^ cell population plays a crucial role in mineralized fibrocartilage formation (Dyment et al., 2015; Schwartz et al., 2015). Recent single-cell RNA-seq analyses have identified distinct cell subpopulations in fibrocartilaginous entheses (Fang et al., 2022). Therefore, it is increasingly important to validate specific cell populations at the protein level and determine the spatiotemporal localization of each cell population within the tissue.

The Achilles tendon, the body’s strongest and largest tendon, connects the soleus and gastrocnemius muscles to the calcaneus. At birth, the Achilles tendon, positive for tenomodulin (Tnmd), attaches to the calcaneus and consists of hyaline cartilage positive for chondromodulin (Sugimoto et al., 2013a). The fibrocartilaginous enthesis of the Achilles tendon develops postnatally in response to mechanical stimuli (Benjamin and Ralphs, 1998; Benjamin et al., 2006). Fibrochondrocytes rapidly mature to mineralize their surrounding matrix through Hedgehog signaling activation (Dyment et al., 2015; Schwartz et al., 2015). Unlike mineralized hyaline cartilage, except for the bottom zone of articular cartilage, mineralized fibrocartilage remains avascular and is not replaced by bone throughout life (Dyment et al., 2015). Injuries disrupt the gradual mineral transition, leading to decreased mechanical performance at the load-bearing interface. The healing process deviates from developmental processes, resulting in a lack of functionally graded layers in the fibrocartilaginous enthesis (Ideo et al., 2020). Despite the coordination of intrinsic factors and extrinsic mechanical forces via the tendon in regulating the mechanical tissue integrity of the fibrocartilaginous enthesis, the formation mechanism of such a connection remains uncertain.

Sclerostin, the product of the *Sost* gene, is a secreted protein predominantly expressed in osteocytes, and also in articular hypertrophic chondrocytes (van Bezooijen et al., 2004; Poole et al., 2005; van Bezooijen et al., 2009). Sclerostin antagonizes canonical Wnt signaling and several BMP responses (Li et al., 2005; Krause et al., 2010). It acts as a negative regulator of bone formation and promotes bone resorption (Baron and Kneissel, 2013). *Sost* deficiency leads to sclerostenosis, Van Buchem’s disease, and autosomal recessive disorders (Brunkow et al., 2001; van Bezooijen et al., 2009). In this study, we investigated the functional role of sclerostin in the postnatal development of the fibrocartilaginous enthesis of the Achilles tendon. For immunostaining and atomic force microscopy (AFM) analysis, we utilized Kawamoto’s film method with cryofilms for thin fresh frozen sections from undecalcified hard tissues (Kawamoto, 2003; Takimoto et al., 2015; Kawamoto and Kawamoto, 2021). This method allows more antibodies to work on fresh, non-decalcified sections without compromising the epitope using excess heat or organic solvents. Persistent sclerostin expression was detected in the mineralized mature fibrochondrocyte layer adjacent to the subchondral bone. In *Scx*-deficient mice, with decreased mechanical loading due to defective tendon formation (Killian and Thomopoulos, 2016; Yoshimoto et al., 2017; Shukunami et al., 2018), both fibrocartilage and hyaline cartilage development were impaired, and sclerostin expression markedly decreased. Loss of *Sost* resulted in increased bone mineral density in the subchondral bone and mineralized fibrocartilage. AFM analysis revealed significantly higher stiffness in fibrocartilage in *Sost*-deficient mice. Therefore, sclerostin in mature mineralized fibrochondrocytes modulates the degree of mineralization and the stiffness profile of the fibrocartilaginous enthesis for mechanical tissue integrity.

## 2 Materials and methods

### 2.1 Animals and embryos

C57BL/6 mice were purchased from CLEA Japan, Inc. and bred for experiments. The generation of *ScxGFP* transgenic and *Scx* ^*Δ11/Δ11*^ strains has been previously reported (Sugimoto et al., 2013b; Shukunami et al., 2018). For detection of the activation of WNT/β-catenin pathway signaling, *Axin2-CreERT2* (JAX stock #018867) and *Rosa-CAG-LSL-tdTomato* (*RosaTomato*) obtained from the Jackson Laboratory were bred with *Runx2GFP* or *Col1GFP* mice (Yang et al., 2019; Mizoguchi, 2024). All animal experimental protocols were approved by the Animal Care Committee of the Institute for Life and Medical Sciences, Kyoto University, and the Committee of Animal Experimentation, Hiroshima University, or Tokyo Dental College, and conformed to the institutional guidelines for vertebrate studies.

### 2.2 Generation of TALEN-mediated *Sost*-deficient mice

TALEN plasmids were constructed using the Platinum Gate TALEN Kit (Kit #1000000043, Addgene, Cambridge, MA, United States of America), as described previously (Sakuma et al., 2013). To prepare TALEN mRNA, TALEN plasmids *mSostTALEN-B-L* and *mSostTALEN-B-R* were linearized with SmaI and purified by phenol-chloroform extraction. *mSostTALEN-B-L* and *-R* mRNAs were synthesized, and a polyA tail was added using the mMESSAGE mMACHINE T7 ULTRA Kit (Ambion, Austin, TX, United States of America), according to the manufacturer’s instructions. After purification using the MEGAclear kit (Ambion, Austin, TX, United States of America), *mSostTALEN-B-L* and *mSostTALEN-B-R* mRNAs were microinjected into the cytoplasm of fertilized eggs obtained from C57BL/6 mice. The injected eggs were then transferred to the oviducts of pseudopregnant surrogate ICR female mice. Genomic DNA was extracted from the tail tips of the founder mice. A 444-bp fragment of exon 1, including recognition sites for TALENs, was amplified by PCR using primers (*Sost_GTF1*:5′-AAGGCAACCGTATCTAGGCTGG-3′; *Sost_GTR1*:5′-CCT​CCA​GGT​TCT​AAT​GCT​GTG​CTA​G-3′). The amplified fragments underwent direct sequencing using a BigDye Terminator Cycle Sequencing kit and an ABI 3100 Genetic Analyzer (Applied Biosystems, Foster City, CA, United States of America). Genomic DNA extracted from mouse ear pieces was subjected to PCR using a specific primer set (*Sost_GTF3*:5′-CCCGTGCCTCATCTGCCTACTTG-3’; *Sost_GTR2*:5′-TCTTCATCCCGTACCTTTGGC-3′), and the amplified fragments were analyzed using MultiNA (SHIMADZU).

### 2.3 Western blot analysis

The tibia was dissected from *Sost* ^*Δ26/+*^ and *Sost* ^*Δ26/Δ26*^ male mice at P120. The isolated tissue was homogenized in RIPA buffer containing Halt Protease Inhibitor Cocktail (Thermo Fisher Scientific) and Halt Phosphatase Inhibitor Single-Use Cocktail (Thermo Fisher Scientific). The concentrations of the tissue extracts were quantified using a BCA protein assay kit (Takara). Samples and Precision Plus Protein Dual Xtra Prestained protein Standards (Bio-Rad Laboratories) were electrophoresed on a 10% TGX Stain-Free gel (Bio-Rad Laboratories) and transferred to a polyvinylidene fluoride membrane (Bio-Rad Laboratories) using a Trans-Bio Turbo Transfer System (Bio-Rad Laboratories). The membrane was incubated with an anti-mouse SOST/Sclerostin (R&D Systems, AF1589, 1:500) antibody in Bullet Blocking One (Nacalai Tesque) and then an anti-GAPDH antibody (FUJIFILM Wako Pure Chemical Corporation, 1:2000), followed by incubation with horseradish peroxidase-conjugated anti-goat IgG or anti-mouse IgG. Peroxidase activity was detected using the SuperSignal West Pico Chemiluminescent Substrate (Thermo Fisher Scientific).

### 2.4 Immunostaining

Anesthetized mice were perfused with 4% paraformaldehyde in phosphate-buffered saline (PFA/PBS) containing 16.6% or 20% sucrose. The specimens were fixed in 4% PFA/PBS containing 16.6% or 20% sucrose at 4°C for 1–3 h, embedded in SCEM or SCEM-L1 (Section-Lab). Undecalcified frozen sections at a thickness of 4 µm were obtained according to Kawamoto’s film method using TC-65 (Leica Microsystems) or SL-T35 (UF) (Section-Lab) tungsten carbide blades, and Cryofilm type 2C (9) or Cryofilm type 3 (16UF) (Section-Lab) (Kawamoto, 2003; Kawamoto and Kawamoto, 2021). After washing with ethanol and PBS, the sections were fixed in 4% PFA/PBS for 5 min and/or decalcified with 0.25 M ethylenediaminetetraacetic acid (pH 8.0). For the detection of GFP, Sox9, Sclerostin, CD31, Ocn, Col1, and Col2 (for P14), sections were treated with hyaluronidase (Sigma–Aldrich) at 37°C. Sections for the detection of Col2 (for P28) were treated with 1 μg/mL of protein kinase K. The sections were fixed in 4% PFA/PBS. The sections treated with hyaluronidase were boiled in 10 mM sodium citrate buffer (pH 6.0). For the detection of GFP, Sox9, Col10, Sclerostin, Ocn, and CD31, the sections were permeabilized in 0.2% Triton X-100 in PBS. The sections were incubated with primary antibodies for 16 h or overnight, washed, and then incubated with the appropriate secondary antibodies conjugated with Alexa Fluor 488 or 594 (Life Technologies, Cell Signaling Technology). Nuclei were counterstained with 4′,6-diamidino-2-phenylindole (DAPI) (Sigma–Aldrich). The primary antibodies used were anti-GFP (Nacalai Tesque, GF090R; 1:1000), anti-Sox9 (MILLIPORE, AB5535; 1:800), anti-Col1 (ROCKLAND, 600-401-103-0.1; 1:500), anti-Col2 (ROCKLAND, 600-401-104-0.1; 1:500), anti-Col10 (Abcam, ab260040; 1:250), anti-Mouse SOST/Sclerostin (R&D Systems, AF1589; 1:500), anti-Ocn (Takara, M173; 1:800), and anti-CD31 (BD, 553370; 1:2000). Images were captured using a Leica DMRXA microscope equipped with a Leica DFC310 FX camera (Leica Microsystems).

### 2.5 *In vivo* labeling of bone with fluorochromes

Intraperitoneal injection of Calcein (10 μg/g body weight) (DOJINDO, 348–00434) and Alizarin Complexone (30 μg/g body weight) (TOKYO CHEMICAL INDUSTRY CO., LTD., A3227) diluted in 2% NaHCO_3_ was delivered to mice based on experimental designs. Labeled mice were anesthetized and perfused with 4% PFA/PBS containing 16.6% sucrose and fixed in 4% PFA/PBS containing 16.6% sucrose at 4°C for 2 h. Undecalcified frozen sections at a thickness of 4 µm were obtained according to Kawamoto’s film method (Kawamoto, 2003; Kawamoto and Kawamoto, 2021). Nuclei were counterstained with DAPI (Sigma–Aldrich), and the images were captured under a Leica DMRXA microscope equipped with a Leica DFC310 FX camera (Leica Microsystems).

### 2.6 Histological staining

For undecalcified frozen sections, anesthetized mice were perfused with 4% PFA/PBS containing 16.6% or 20% sucrose and fixed in 4% PFA/PBS containing 16.6% or 20% sucrose at 4°C for 2 or 3 h. Undecalcified frozen sections were stained with 0.05% toluidine blue (TB) solution at pH 4.1 (MUTO PURE CHEMICALS CO., LTD.) for 5 min, tartrate-resistant acid phosphatase (TRAP) staining with a TRAP/alkaline phosphatase (ALP) Stain Kit (FUJIFILM Wako Pure Chemical Corporation) for 30 min, or ALP staining with NBT/BCIP solution (Roche; 1:100) for 15 min, followed by Alizarin red (AR) staining prepared from 1% AR Solution at pH 6.3–6.4 (MUTO PURE CHEMICALS CO., LTD.) for 5 min.

### 2.7 Atomic force microscope-based tissue indentation

Anesthetized male mice were perfused with PBS, and the Achilles tendon entheses were dissected. Specimens were prepared from *Sost* ^*+/+*^ or *Sost* ^*Δ26/Δ26*^ mice perfused with PBS, embedded in SCEM (Section-Lab), frozen in hexane (FUJIFILM Wako Pure Chemical Corporation). Undecalcified frozen sections at a thickness of 20 µm were obtained according to Kawamoto’s film method (Kawamoto, 2003; Kawamoto and Kawamoto, 2021). The cell nuclei were stained with Hoechst 33342. For the AFM-based tissue indentation (Ichijo et al., 2022), a JPK BioAFM NanoWizard 3 (Bruker Nano GmbH) was employed. The AFM system was mounted on a bright-field fluorescence microscope (IX81; Evident Co.). AFM cantilevers (TL-CONT; spring constant 0.2 N/m; Nanoworld AG) were modified with glass beads with a diameter of 10 μm and calibrated using the thermal noise method (Butt and Jaschke 1995). To identify the tendon, fibrocartilage, and bone regions, Hoechst-stained tissue sections were observed by IX81 microscope. In particular, AR-stained sections from the same tissue were used to classify the unmineralized and mineralized fibrocartilage regions. For AFM-based indentation, the piezo displacement speed and the sampling rate were set as 3 μm/s and 4,000 Hz, respectively. The obtained indentation force (*F*) versus depth (*h*) curve was smoothed using a moving average of 10 datum points before and after each averaging point. Stiffness [nN/μm] was estimated by linear regression for the (*F*, *h*) datum points within an indentation depth range of 0 nm ≤ *h* ≤ 50 nm.

### 2.8 Skeletal imaging by microcomputed tomography analysis

Mice anesthetized at P28 and P120 were perfused with 4% PFA/PBS or PBS and soaked in 99.5% ethanol (Wako, 057–00456). Mice were analyzed by InspeXio SMX-90CT Plus (SHIMADZU) in 99.5% ethanol (Wako, 057–00456) with a 90 kV source voltage and 110 µA source current and a resolution of 0.026 mm/pixel (*n* = 4 or 6 biological replicates for each group). Two- and three-dimensional reconstructions were performed using Amira 3D software version 2021.1 (Thermo Fisher Scientific). Bone mineral density was calculated from the equation derived from the least-squares method with five plots using hydroxyapatite phantom (RATOC, No06-U5D1mmH) with 100, 200, 300, 400, and 500 mg/cm^3^ separately scanned on the same day under the same conditions as the samples.

### 2.9 Statistics

All statistical analyses were performed using Microsoft Excel or GraphPad Prism, version 9 (GraphPad Software, LLC). Data are presented as mean ± SD. Comparisons were performed using the unpaired t-test (for cortical bone thickness and bone mineral density), unpaired t-test with Welch’s correction (for trabecular bone volume/tissue volume), or Mann–Whitney *U* test (for AFM-based tissue indentation) to determine significance between groups. The level of significance was set at *p* < 0.05.

## 3 Results

### 3.1 Expression of sclerostin in mature fibrochondrocytes of the mineralized fibrocartilage

**FIGURE 1:**
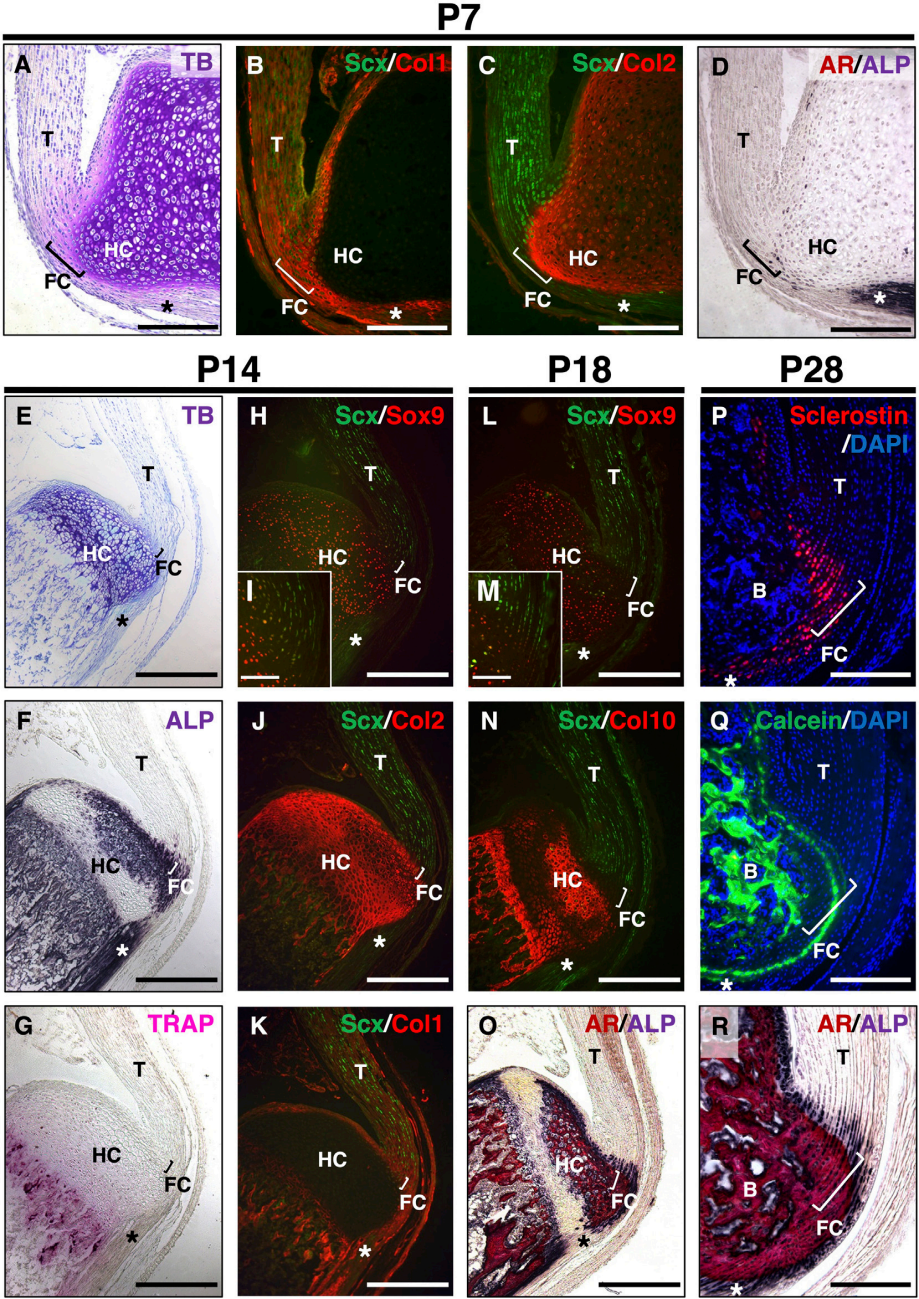
Postnatal development of entheseal fibrocartilage. Undecalcified frozen sections of the Achilles tendon enthesis were prepared from *ScxGFP* Tg mice at P7 **(A–D)**, P14 **(E–K)**, or P18 **(L–O)**. Sagittal sections were processed for staining of TB **(A,E)**, ALP **(D,F,O,R)**, TRAP **(G)**, and AR **(O,R)** or immunostaining with antibodies against GFP for Scx expression (green) **(B,C,H–N)** and Sox9 (red) **(H,I,L,M)**, Col2 (red) **(C,J)**; Col1 (red) **(B,K)**; or Col10 (red) **(N)**. The insets **(I,M)** show high-magnification images of the fibrocartilage immunostained with antibodies against GFP (green) and Sox9 (red). Undecalcified frozen sections of the Achilles tendon enthesis at P28 were prepared from wild-type mice administered Calcein at P21 and P27. Immunostaining with sclerostin (red) is shown in **(P)**, and mineral apposition is indicated by Calcein labeling (green) in **(Q)**. Nuclei were stained with DAPI **(P,Q)**. AR and ALP (AR/ALP) staining is shown **(R)**. Square brackets indicate the fibrocartilage of the enthesis. Asterisks indicate the plantaris tendon. Data are representative of at least three same-week-old mice per group. Abbreviations: T, tendon; FC, fibrocartilage; HC, hyaline cartilage; B, bone. Scale bars: 200 µm **(A–D,I,M,P–R)**, 400 µm **(E–H,J–L,N,O)**.

In hyaline cartilage, Sox9^+^ chondrocytes produce Col2 and Acan and then mature to become hypertrophic chondrocytes, synthesizing Col10 prior to mineralization (Kozhemyakina et al., 2015). Using Kawamoto’s film method for sectioning undecalcified hard tissues, we compared the expression of these cartilage markers in the calcaneus and their insertion sites of the Achilles tendon by immunostaining (Figure 1). Expansion of the unmineralized fibrocartilage and underlying hyaline cartilage at P7 was visualized using TB staining (Figure 1A). Col1 was co-expressed with Scx in the tendon and fibrocartilage (Figure 1B). Col2 was detected in both the epiphyseal hyaline cartilage and fibrocartilage, whereas Scx was expressed in the upper portion of the Col2^+^ fibrocartilage near the tendon (Figure 1C). Only a small number of ALP^+^ cells were observed at the junction between the unmineralized fibrocartilage and epiphyseal hyaline cartilage (Figure 1D). At P14, hypertrophic chondrocytes were observed in the epiphyseal hyaline cartilage beneath the fibrocartilage (Figure 1E). ALP activity was high in osteoblasts, fibrochondrocytes, and chondrocytes, except in the resting hyaline cartilage (Figure 1F). Chondroclasts/osteoclasts positive for TRAP were found in the mineralized hypertrophic cartilage of the growth plate and primary spongiosa (Figure 1G). Intense Col2 staining was detected in Sox9^+^ cartilage and cartilage remnants around the chondro-osseous junction (Figures 1H–J), whereas Col1 was co-expressed with Scx in the fibrocartilage, tendon, and primary spongiosa (Figure 1K). At P18, Sox9 was expressed in proliferating and resting chondrocytes (Figures 1L,M). In the hyaline cartilage, hypertrophic/mineralized chondrocytes strongly expressed Col10, whereas its expression in the mineralized fibrocartilage was low (Figures 1N,O). Therefore, mineralizing chondrocytes are divided into two distinct groups: Col2^+^/Col10^+++^/Col1^–^ hypertrophic chondrocytes in the epiphyseal hyaline cartilage and Col2^++^/Col10^+^/Col1^++^ fibrochondrocytes. We also observed that sclerostin was expressed in mature fibrochondrocytes at P28 (Figure 1P). Calcein labeling and ALP/AR staining revealed that the mineralization front consisting of ALP^+^ cells extended towards the midsubstance of the Achilles tendon (Figures 1Q,R).

**FIGURE 2:**
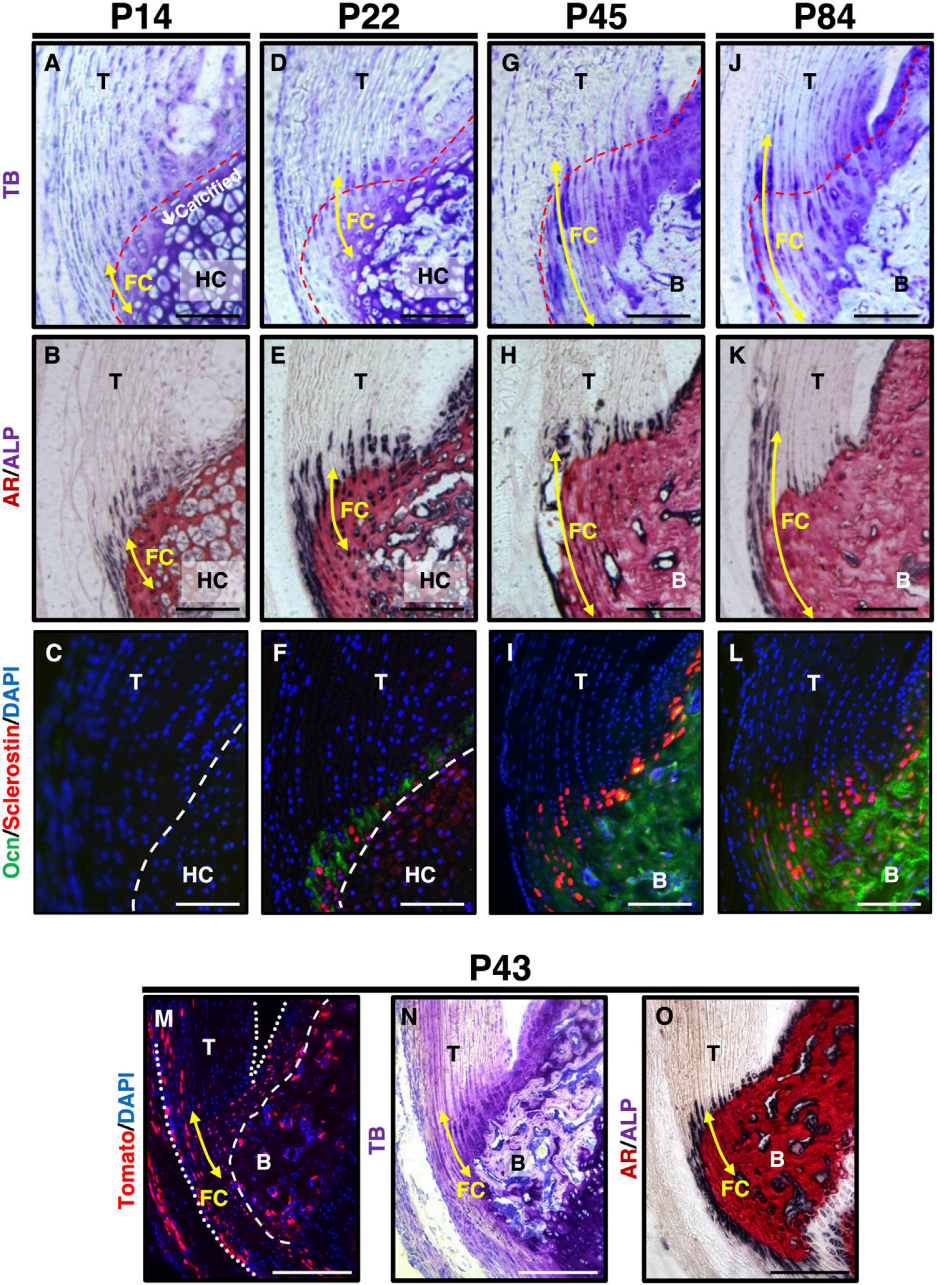
Expression of sclerostin in mature fibrochondrocytes of the Achilles tendon enthesis. (**A–L**) Undecalcified frozen sections of the Achilles tendon enthesis were prepared from *ScxGFP* Tg mice at P14 **(A,B)**, P22 **(D,E)**, P45 **(G,H)**, or P84 **(J,K)** and wild-type mice at P14 **(C)**, P22 **(F)**, P45 **(I)**, or P84 **(L)**. Sagittal sections were stained with TB **(A,D,G,J)**, AR/ALP **(B,E,H,K)** and were processed for immunostaining of Ocn (green) and Sclerostin (red) **(C,F,I,L)**. The nuclei were stained with DAPI (blue). **(M–O)** Undecalcified frozen sections of the Achilles tendon enthesis were prepared from a *Axin2CreERT2;RosaTomato*;*Runx2GFP* at P43 after 5 days of tamoxifen diet. Cre-mediated Tomato expression was detected as red fluorescence **(M)**. Sagittal sections were stained with TB (N) or AR/ALP **(O)**. The nuclei were stained with DAPI (blue). Yellow arrows indicate fibrocartilage. Red dashed line in TB staining indicates the tidemark between the unmineralized and mineralized fibrocartilage. White dashed line in immunostaining indicates the boundary between fibrocartilage and hyaline cartilage. The Achilles and superficial digital flexor tendons are enclosed by white dotted lines in **(M)**. Data are representative of at least three same-week-old mice per group. Abbreviations: T, tendon; FC, fibrocartilage; HC, hyaline cartilage; B, bone. Scale bars: 100 µm in **(A–L)** and 200 µm in **(M–O)**.

We examined the expression profile of sclerostin during the fibrocartilaginous enthesis (Figure 2). At P14, most fibrochondrocytes were present in the mineralized region adjacent to the mineralized hyaline cartilage, which consisted of hypertrophic chondrocytes (Figures 2A,B). ALP^+^ cells were observed in both the unmineralized and mineralized regions (Figure 2B). Osteocalcin (Ocn) and sclerostin were not detected in either fibrocartilage or hyaline cartilage at P14 (Figure 2C). At P22, a secondary ossification center appeared in the epiphysis, where the mineralized hyaline cartilage was invaded by blood vessels and gradually replaced by bone (Figures 2D,E). More intense ALP staining was observed in the unmineralized and mineralized fibrocartilage, hyaline cartilage, and subchondral bone (Figure 2E). Sclerostin^+^ cells were observed in the Ocn-expressing mineralized fibrocartilage at P22 (Figure 2F). The size of fibrochondrocytes in the mineralized fibrocartilage was much smaller than that of the hypertrophic chondrocytes in the mineralized hyaline cartilage (Figures 2B,E). By P45, the epiphyseal mineralized hyaline cartilage was replaced with bone, and the entheseal mineralized fibrocartilage expanded further (Figures 2G,H). More sclerostin-expressing cells were observed in the OCN-deposited mineralized fibrocartilage (Figure 2I). At P84, the expansion of the unmineralized fibrocartilage above the mineralized fibrocartilage was more evident, in association with a decrease in ALP^+^ cells (Figures 2J,K); however, the plantaris tendon was still ALP^+^ (Figure 2K). Sclerostin expression largely overlapped with Ocn expression in the fibrochondrocytes (Figure 2L). In the epiphysis, the hyaline cartilage was completely replaced by bone (Figures 2H, K), and rapid mineralization of the fibrocartilage without significant cellular hypertrophy was followed by the expansion of the unmineralized fibrocartilage (Figures 2J,K). The expansion of mineralized fibrocartilage is guided by ALP^+^ cells at the mineralization front. This was followed by the expansion of unmineralized fibrocartilage after a decrease in the number of ALP^+^ cells. We then examined the activation of canonical Wnt signaling in the fibrocartilaginous enthesis using 4-week old *Axin2-CreERT2;RosaTomato* mice with *Runx2GFP* or *Col1GFP* reporters. For induction of Cre-recombinase, tamoxifen diet was given for 5 days and then sacrificed at P43 (Figures 2M–O) or P45 (Supplementary Figure S1). Axin2-lineage cells visualized by Tomato expression were found in fibrocartilage as well as subchondral bone and tendon (Figures 2M–O and Supplementary Figures S1B–D). These results suggest that sclerostin is an excellent marker for mature fibrochondrocytes located in the mineralized fibrocartilage adjacent to the subchondral bone, and that activation of canonical Wnt signaling occurs in fibrochondrocytes of the developing enthesis.

### 3.2 Defective fibrocartilage formation in association with a decrease in sclerostin expression in the Achilles tendon enthesis of *Scx*-deficient mice

The Achilles tendon of *Scx*-deficient mice were defective and negative for Tnmd, a mature tenocyte marker (Shukunami et al., 2006; Yoshimoto et al., 2017; Shukunami et al., 2018). The loss of *Scx* leads to defective tendon and enthesis formation, resulting in impaired mechanical outcomes (Killian and Thomopoulos, 2016; Yoshimoto et al., 2017). We analyzed the changes in sclerostin expression together with cartilage and blood vessel markers in the defective Achilles tendon enthesis of *Scx* ^*Δ11/Δ11*^ mice (Shukunami et al., 2018).

**FIGURE 3:**
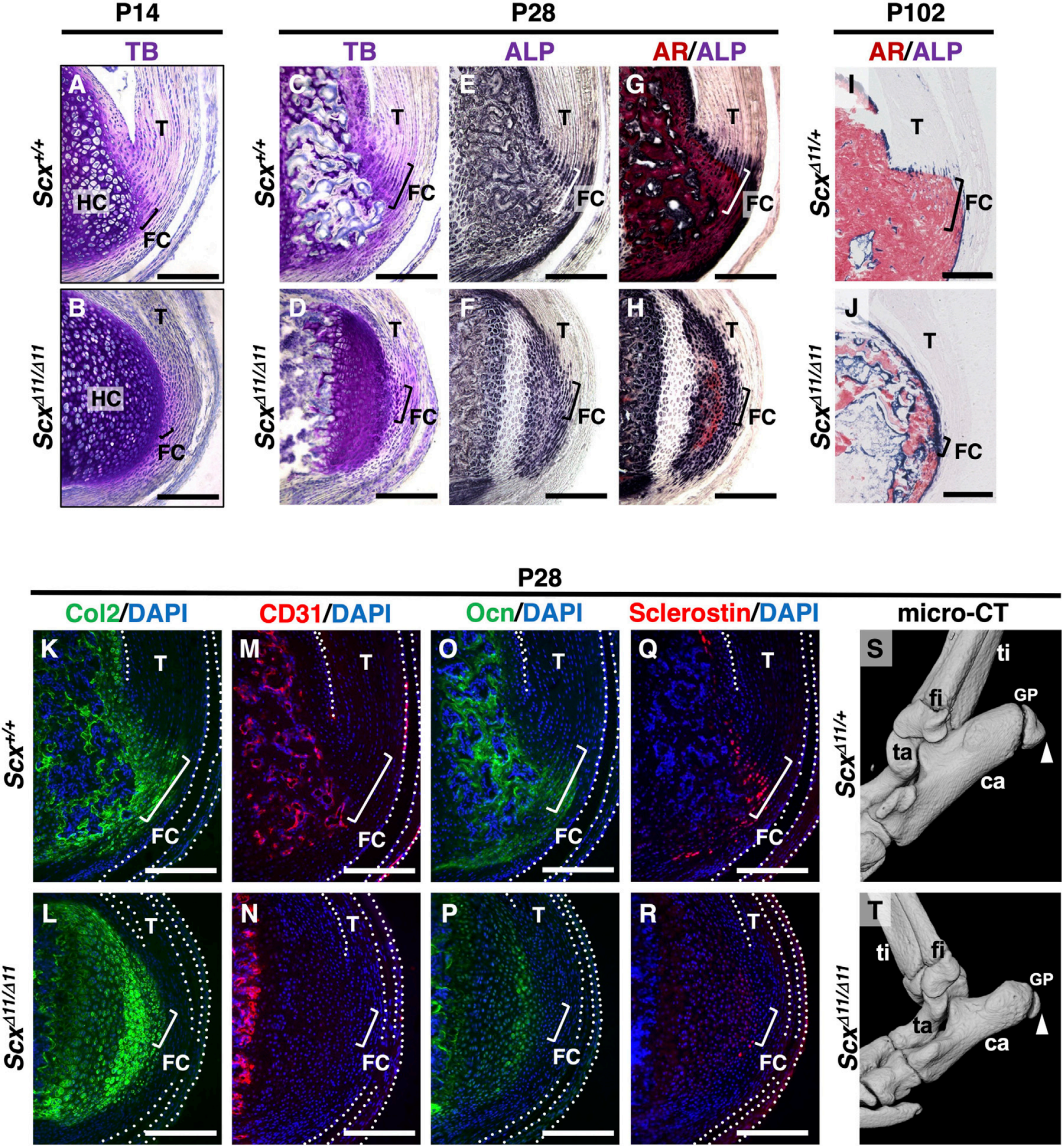
Defective fibrocartilage formation in *Scx*-deficient mice. Undecalcified frozen sections of the Achilles tendon enthesis were prepared from *Scx* ^*+/+*^ **(A)** and *Scx* ^*Δ11/Δ11*^ **(B)** mice at P14 **(A,B)**, or age-matched *Scx* ^*+/+*^ **(C,E,G,K,M,O,Q)** and *Scx* ^*Δ11/Δ11*^ **(D,F,H,L,N,P,R)** mice at P28, or *Scx* ^*Δ11/+*^ **(I)** and *Scx* ^*Δ11/Δ11*^ **(J)** mice at P102. Sagittal sections were stained with TB **(A–D)**, ALP **(E,F)**/AR **(G,H)**, or immunostained with antibodies against Col2 (green) **(K,L)**, CD31 (red) **(M,N)**, Ocn (green) **(O,P)**, or Sclerostin (red) **(Q,R)**. Nuclei were stained with DAPI (blue) **(K–R)**. The Achilles and superficial digital flexor tendons are enclosed by white dotted lines in **(K–R)**. The square brackets indicate fibrocartilaginous entheses. Micro-CT images of *Scx* ^*Δ11/*+^ **(S)** and *Scx* ^*Δ11/Δ11*^ **(T)** mice at P28. White arrowheads in **(S,T)** indicate the Achilles tendon enthesis. Data are representative of at least three same-week-old mice per group except for two independent heterozygotes or homozygotes at P102. Abbreviations: T, tendon; FC, fibrocartilage; HC, hyaline cartilage; ti, tibia; ca, calcaneus; fi, fibula; ta, talus; GP, growth plate. Scale bars: 200 µm **(A–R)**.

At P14 in *Scx* ^*+/+*^ mice, the columnar fibrochondrocytes of the enthesis were small, whereas the chondrocytes in the epiphyseal hyaline cartilage of the calcaneus became hypertrophic (Figure 3A). Metachromatic staining with TB was weak in fibrocartilage and strong in hyaline cartilage (Figures 3A,B). In association with defective formation of the Achilles tendon in *Scx* ^*Δ11/Δ11*^ mice, both fibrocartilaginous enthesis formation and maturation of epiphyseal hyaline cartilage were defective (Figures 3A,B).

At P28, the Achilles tendon enthesis was convex and mineralized in *Scx* ^*+/+*^ mice, but rounded and unmineralized in *Scx* ^*Δ11/Δ11*^ mice (Figures 3C–H). Fibrochondrocytes in *Scx* ^*+/+*^ mice were arranged in a column along the collagen fibers connected to the Achilles tendon (Figure 3C). However, the layer of fibrocartilage with irregularly aligned fibrochondrocytes was thin and unmineralized in *Scx* ^*Δ11/Δ11*^ mice (Figure 3D). In *Scx* ^*+/+*^ mice, the replacement of mineralized fibrocartilage with bone was observed in the secondary ossification center (Figures 3C,E,G). In contrast, in *Scx* ^*Δ11/Δ11*^ mice, cellular hypertrophy and mineralization of epiphyseal hyaline cartilage were delayed, and vascular invasion did not occur (Figures 3D,F,H). At P102, enthesis and epiphyseal bone formation were complete in *Scx* ^*Δ11/+*^ mice (Figure 3I), while the immature epiphysis was covered with thin ALP^+^ cells in *Scx* ^*Δ11/Δ11*^ mice (Figure 3J).

We then analyzed Sclerostin, Ocn, CD31 (a marker of vascular endothelial cells), and Col2 localization in *Scx* ^*+/+*^ or *Scx* ^*Δ11/Δ11*^ mice at P28 (Figures 3K–R). Wild-type sclerostin^+^ fibrochondrocytes co-expressed Ocn in mineralized entheses that were negative for CD31 (Figures 3M,O,Q) but positive for Col2 (Figure 3K). In *Scx* ^*Δ11/Δ11*^ mice, vascular invasion did not occur in the epiphyseal cartilage, and sclerostin was faintly co-expressed with Ocn and Col2 (Figures 3L,N,P,R).

Micro-CT imaging at P28 revealed that the enthesis of *Scx* ^*Δ11/Δ11*^ mice was round and undermineralized compared with *Scx* ^*Δ11/+*^ mice (Figures 3S,T). These results suggest that mechanical stimulation is essential for the proper development of fibrocartilaginous enthesis, and that sclerostin expression in the avascular mineralized fibrocartilage is closely associated with fibrochondrocyte maturation.

### 3.3 Increased bone mineral density and higher stiffness in the fibrocartilaginous enthesis of *Sost* ^*Δ26/Δ26*^ mice

**FIGURE 4:**
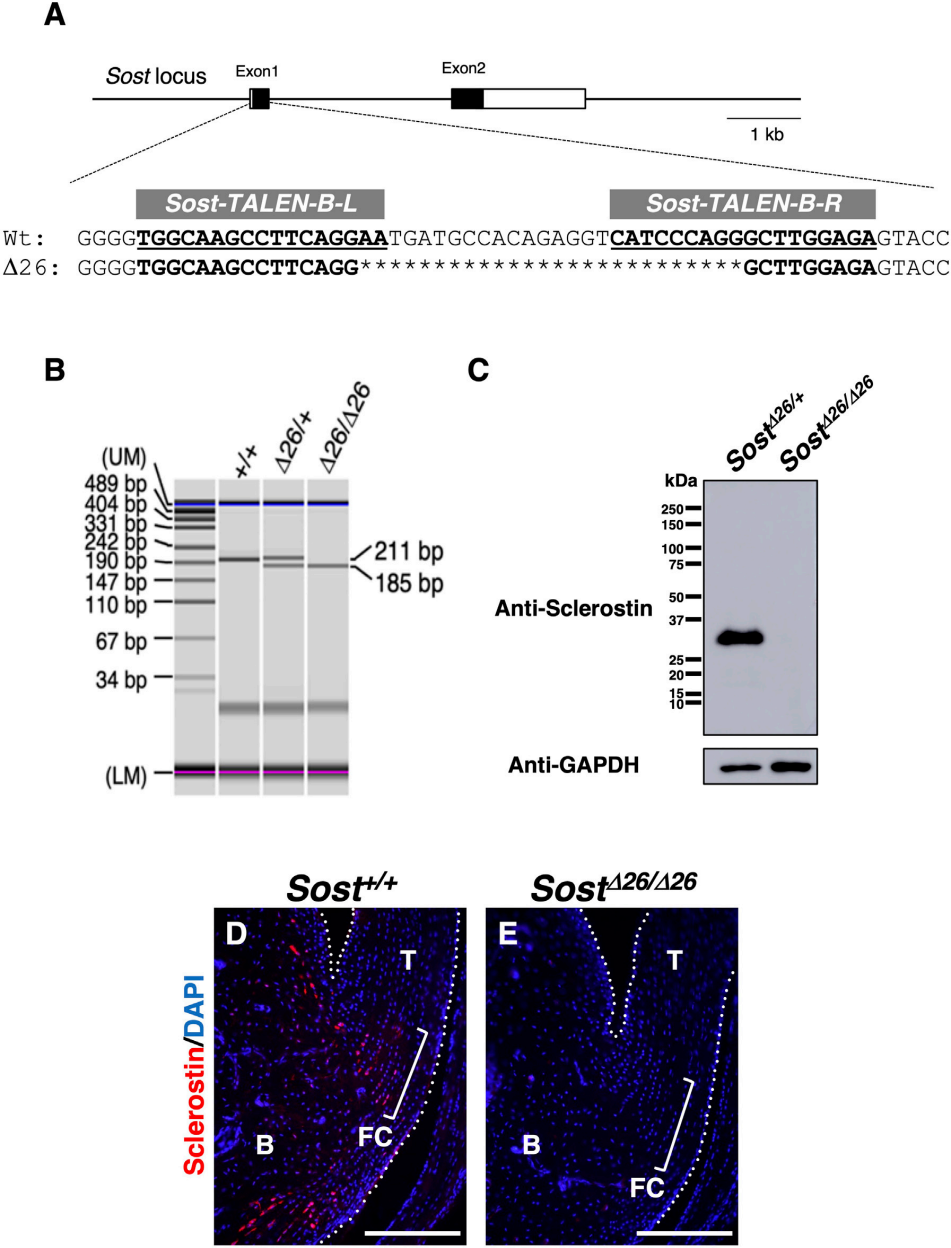
Generation of *Sost*-deficient mice. **(A)** Genomic structures of *Sost* and TALEN target sequences at the mouse *Sost* locus. Left (L) and right (R) binding regions of *mSost-TALEN-B* are indicated in bold and underlined text. Sequences of *Sost* ^*+/+*^ and founder mice generated by microinjection of *mSost-TALEN-B-R/L* mRNAs. Nucleotide deletions are indicated by asterisks. **(B)** Genotyping PCR was performed using DNA from ear pieces with the primers described in the Materials and Methods. The targeted *Sost* ^*Δ26*^ allele (185-bp) and wild-type allele (211-bp) were distinguished. **(C)** Western blotting was performed to detect sclerostin with a molecular weight of ∼28 kDa in the tibial extract of *Sost* ^*Δ26/+*^ and *Sost* ^*Δ26/Δ26*^ mice at P120. GAPDH was detected as a band with a molecular weight of ∼37 kDa in each extract. **(D)** Sagittal sections prepared from *Sost* ^*+/+*^ and *Sost* ^*Δ26/Δ26*^ mice at P90 were immunostained with an antibody against sclerostin (red). The Achilles and superficial digital flexor tendons are enclosed by white dotted line. Square brackets indicate the fibrocartilage of the enthesis. Data are representative of three age-matched mice. Abbreviations: T, tendon; FC, fibrocartilage; B, Bone. Scale bars: 200 µm **(D,E)**.

To elucidate the *in vivo* role of sclerostin in the fibrocartilaginous enthesis, we investigated *Sost*-deficient mice generated using Platinum TALENs (Sakuma et al., 2013) (Figure 4; Supplementary Figure S2). We designed the TALEN recognition sequences to be within exon 1 of the *Sost* locus (Figure 4A; Supplementary Figure S2A), so that most of sclerostin would be lost due to a frameshift mutation causing a premature stop codon after creation of a double stranded break by TALENs. Of 40 newborn mice obtained, 16 pups with deletion mutation and 1 pup with an insertion mutation were identified by genotyping and direct sequencing of the amplified DNA. We have established two lines each with a 26-base pair (bp) or 2-bp deletion, both resulting in frameshift causing a premature stop codon shortly downstream (Figure 4A and Supplementary Figures S2A,B, S3A,B). For genotyping of a line with a 26-bp, the wild-type allele corresponds to the upper 211-bp band and the mutant allele to the lower 185-bp band (Figure 4B). Loss of sclerostin expression in *Sost* ^*Δ26/Δ26*^ mice at P120 was confirmed by Western blotting (Figure 4C; Supplementary Figure S4). Sclerostin localization was observed in the fibrocartilage and bone of *Sost* ^*+/+*^ mice (Figure 4D) but absent in *Sost* ^*Δ26/Δ26*^ mice (Figure 4E) at P90.

**FIGURE 5:**
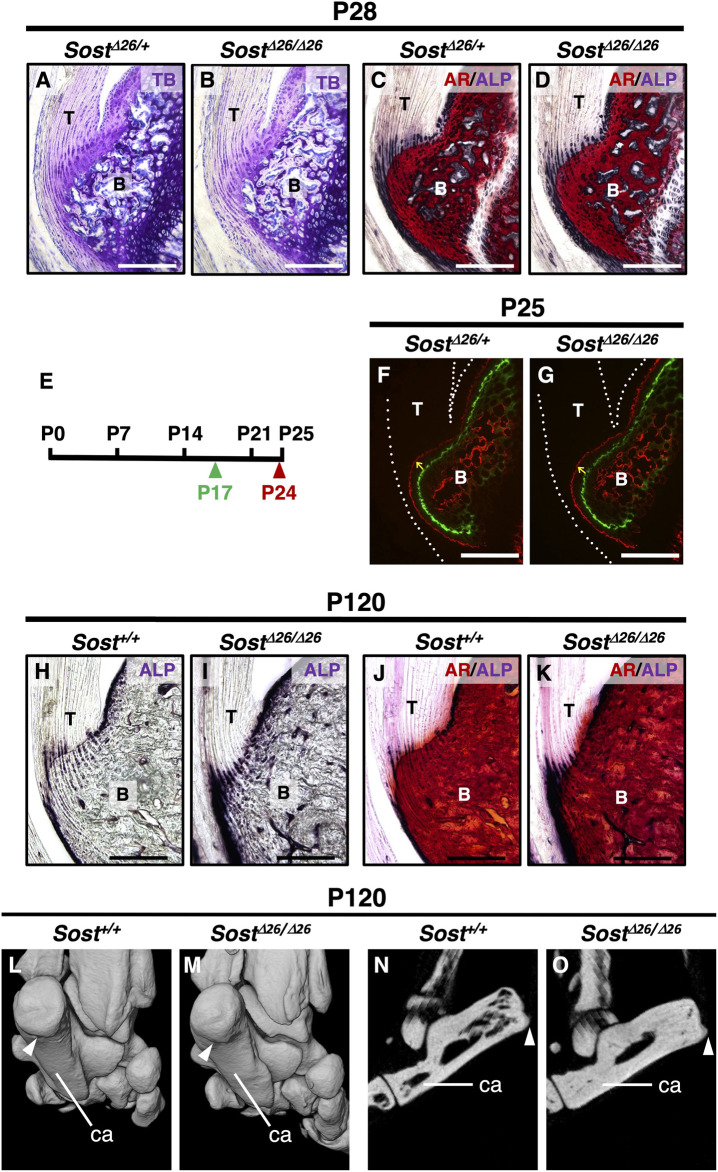
Increased mineralization of the entheseal fibrocartilage and subchondral bone in *Sost-*deficient mice. Undecalcified frozen sections of the Achilles tendon entheses were prepared from *Sost* ^*Δ*26*/+*^ and *Sost* ^*Δ*26*/Δ*26^ mice at P28 **(A–D)**. Sagittal sections were stained with TB **(A,B)** and AR/ALP **(C,D)**. Following the experimental schedule **(E)**, Calcein and Alizarin complexone were administered at P17 (green arrowhead) and P24 (red arrowhead), respectively. Undecalcified frozen sagittal sections of the Achilles tendon enthesis were prepared from *Sost* ^*Δ26/+*^ at P25 **(F)** and *Sost* ^*Δ26/Δ26*^ mice at P25 **(G)**. Yellow arrows denote the direction of fibrocartilage mineralization, and white dotted lines enclose the Achilles tendon **(F,G)**. Undecalcified frozen sections of the Achilles tendon enthesis were prepared from age-matched *Sost* ^+*/+*^ and *Sost* ^*Δ*26*/Δ*26^ mice at P120 **(H–K)**. Sagittal sections were stained with ALP **(H,I)** or AR/ALP **(J,K)**. Micro-CT images of the left heels of age-matched *Sost* ^*+/+*^ **(L,N)** and *Sost* ^*Δ26/Δ26*^ mice **(M,O)** at P120. Three-dimensional views of the left heel are shown **(L,M)** and sagittal plane images are shown **(N,O)**. White arrowheads in **(L–O)** indicate the attachment site of the Achilles tendon to the calcaneus bone. Data are representative of at least three same-week-old mice per group. Abbreviations: T, tendon; B, bone; ca, calcaneus. Scale bars: 200 µm **(A–D,F–K)**.

At P28, the acceleration of replacing mineralized hyaline cartilage with bone was evident in *Sost* ^*Δ26/Δ26*^ mice compared to *Sost* ^*Δ26/+*^mice (Figures 5A–D). To analyze the direction and mineral apposition rate during enthesis formation, we injected two different fluorescent mineralization labels (Calcein and Alizarin complexone) at P17 and P24 in *Sost* ^*Δ26/+*^ or *Sost* ^*Δ26/Δ26*^ mice, euthanizes at P25 (Figure 5E). Mineral apposition occurred from the bottom of the enthesis towards the tendon midsubstance, with the mineral apposition rate in *Sost* ^*Δ26/Δ26*^ mice comparable to that in *Sost* ^*Δ26/+*^ mice (Figures 5F,G). At P120, while the overall number of ALP^+^ cells decreased, more ALP^+^ cells were observed in both the fibrocartilage and bone of *Sost* ^*Δ26/Δ26*^ mice compared to *Sost* ^*+/+*^ mice (Figures 5H–K). Micro-CT images at P120 revealed that the fibrocartilaginous enthesis in *Sost* ^*Δ26/Δ26*^ mice was comparable to *Sost* ^*+/+*^ (Figures 5L,M), yet mineralization was enhanced in *Sost* ^*Δ26/Δ26*^ mice (Figure 5O) compared to *Sost* ^*+/+*^ mice (Figure 5N). Increased bone mineral density was also observed in *Sost* ^*Δ2/Δ2*^ mice compared to *Sost* ^*Δ2/+*^ mice (Supplementary Figure S3).

**FIGURE 6:**
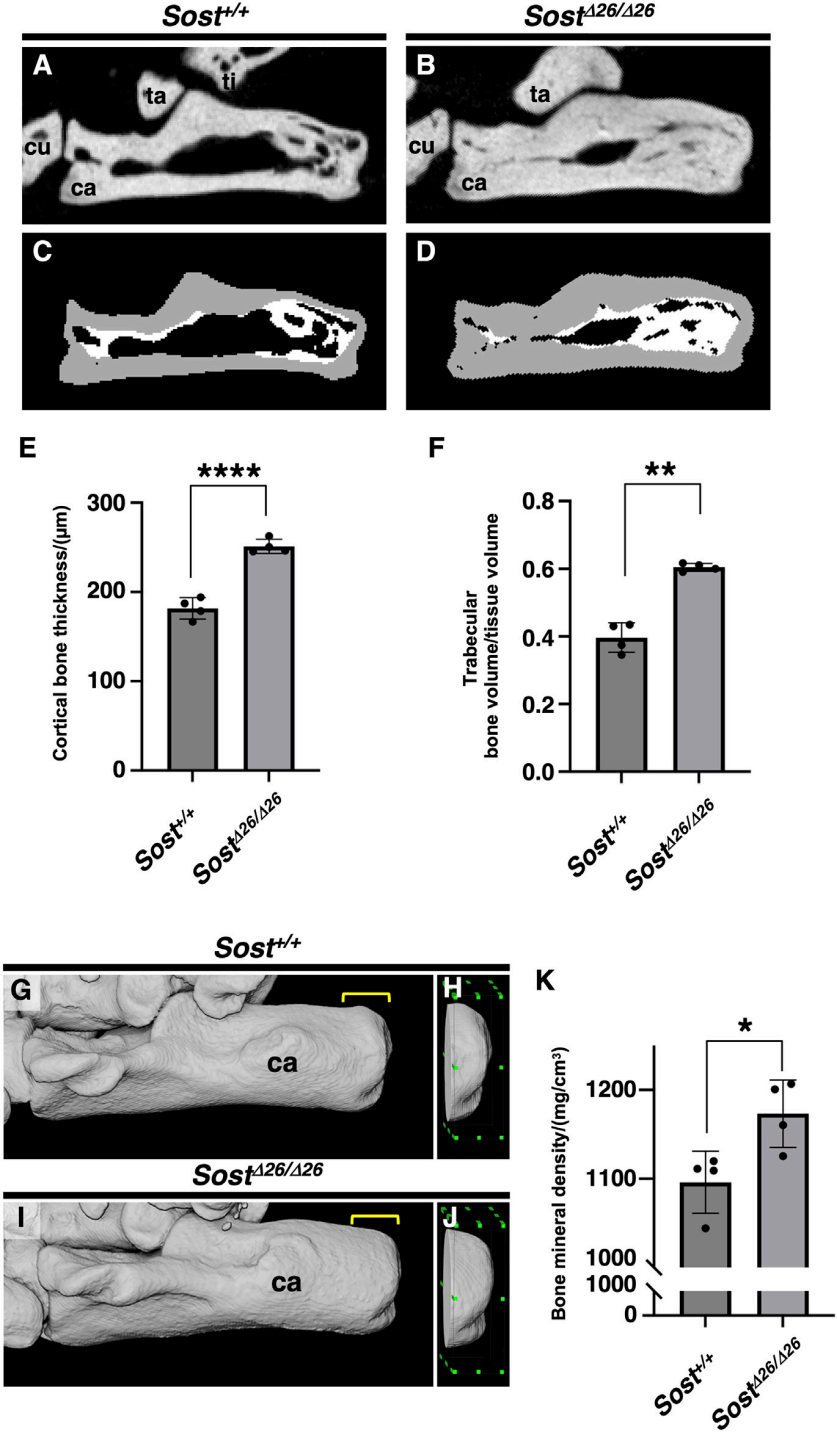
Increased bone volume and mineral density of the calcaneus of *Sost-*deficient mice. Sagittal plane images of the left calcaneus of age-matched *Sost* ^*+/+*^ **(A, C)** and *Sost* ^*Δ26/Δ26*^ **(B, D)** mice at P120. The segmented areas are shown as cortical bone (gray) and trabecular bone (white) **(C, D)**. Cortical bone thickness and trabecular bone volume/tissue volume are shown in **(E)** and **(F)**, respectively. The area 1.8 mm long, 0.5 mm wide, and 1.5 mm high, surrounded by the green dots, is defined as the region of interest (ROI) of the calcaneus for the calculation of bone mineral density in age-matched *Sost* ^*+/+*^ **(G,H)** and *Sost* ^*Δ26/Δ26*^ mice at P120 **(I,J)**. Bone mineral density in the ROI is shown in **(K)**. *n* = 4 biological replicates per group. Data represent mean ± SD. *p < 0.05 (Unpaired t-test), **p < 0.01 (Unpaired t-test with Welch’s t-test), and ****p < 0.0001 (Unpaired t-test). Yellow square brackets in **(G,I)** indicate ROI. Abbreviations: ti, tibia; ca, calcaneus; cu, cuboid; ta, talus.

For quantitative assessment, we scanned the calcaneus of *Sost* ^*+/+*^mice (Figure 6A) and *Sost* ^*Δ26/Δ26*^ mice (Figure 6B) at P120, segmenting the cortex from the trabeculae based on image structure and intensity (Figures 6C,D). Cortical bone thickness (Figure 6E) and trabecular bone volume/tissue volume (Figure 6F) of the calcaneus were significantly higher in *Sost* ^*Δ26/Δ26*^ mice than in *Sost* ^*+/+*^ mice. To analyze the mineralization of the fibrocartilaginous enthesis, we extracted an image of the calcaneus epiphysis, including subchondral bone and mineralized fibrocartilage of *Sost* ^*+/+*^ (Figures 6G,H) or *Sost* ^*Δ26/Δ26*^ mice (Figures 6I,J) at P120, demonstrating significantly increased bone mineral density in *Sost* ^*Δ26/Δ26*^ mice compared to *Sost* ^*+/+*^ mice (Figure 6K).

**FIGURE 7:**
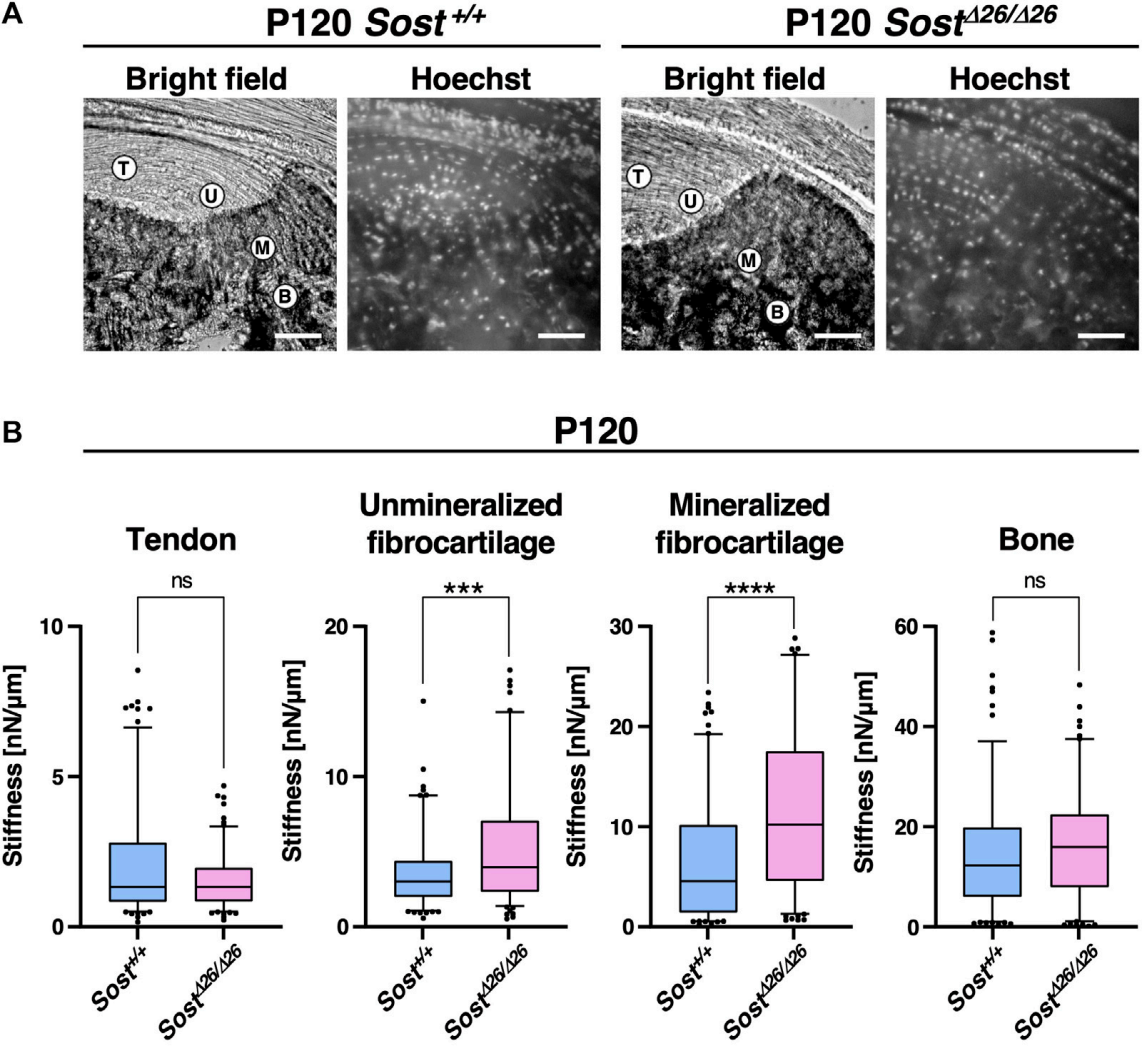
Higher stiffness of the entheseal fibrocartilage in *Sost-*deficient mice. **(A)** For atomic force microscope-based tissue indentation, tendon (T), unmineralized fibrocartilage (UFC; U), mineralized fibrocartilage (MFC; M), and bone **(B)** regions were identified in the cryosections from P120 *Sost* ^+*/*+^ and *Sost* ^*Δ*26*/Δ*26^ mice. Scale bars: 100 µm. **(B)** Box-and-whisker plots of stiffness [nN/µm] for each tissue region. The center line, the box, and the whisker indicate the median value, 25-75 percentile range, and 10-90 percentile range, respectively. *n* = 50 sample points/mouse. ***p < 0.001 and ****p < 0.0001 (Mann–Whitney *U* test). *n* = 3 biological replicates per group.

To assess the stiffness of the enthesis in *Sost* ^*+/+*^ and *Sost* ^*Δ26/Δ26*^ mice at P120, we conducted tissue indentation experiments using AFM (Figure 7). Tendon, Unmineralized/mineralized fibrocartilage and bone were identified using Hoechst staining under a fluorescence microscope (Figure 7A). Both unmineralized and mineralized fibrocartilage exhibited higher stiffness in *Sost* ^*Δ26/Δ26*^ mice, with larger stiffness values, whereas the tendon and bone regions did not show significant differences (Figure 7B).

These findings suggest that *Sost*/sclerostin plays a crucial role in regulating the degree of mineralization, contributing to the modulation of the fibrocartilaginous enthesis gradient and maintaining mechanical tissue integrity.

## 4 Discussion

**FIGURE 8:**
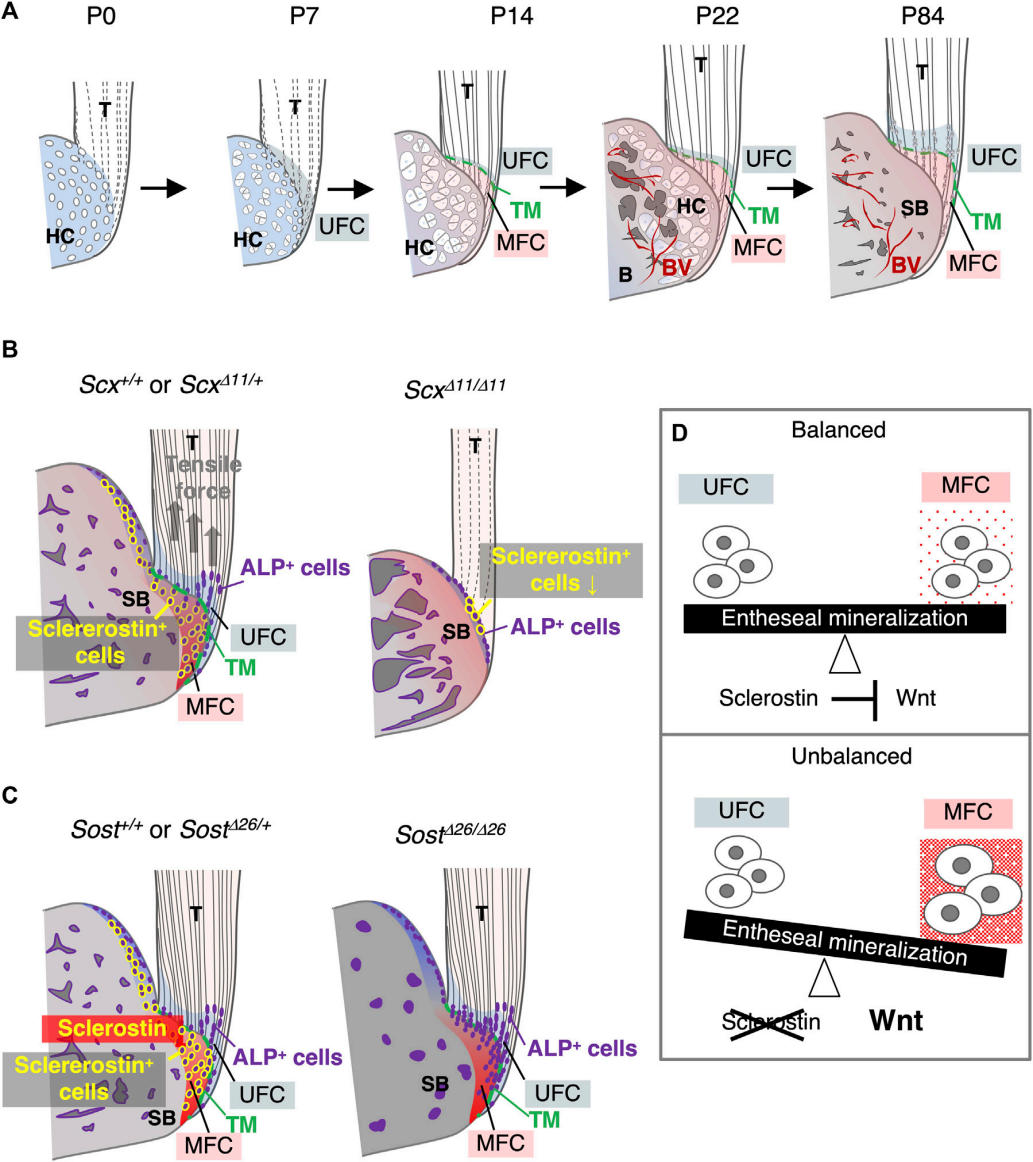
Expression and function of *Sost*/sclerostin in the fibrocartilaginous enthesis. **(A)** Schematic illustration of fibrocartilaginous enthesis formation. Development of the Achilles tendon enthesis at P0, P7, P14, P22, and P84 are presented. **(B)** Schematic illustration of fibrocartilaginous enthesis formation in *Scx* ^*+/+*^ or *Scx* ^*Δ11/+*^ and *Scx* ^*Δ11/Δ11*^ mice. **(C)** Schematic illustration of fibrocartilaginous enthesis formation in *Sost* ^*+/+*^ or *Sost* ^*Δ26/+*^ and *Sost* ^*Δ26/Δ26*^ mice. **(D)** Schematic illustration of the functional role of sclerostin as a modulator of the degree of fibrocartilage mineralization in the enthesis. Abbreviations: T, tendon; HC, hyaline cartilage; MFC, mineralized fibrocartilage; B, bone; BV, blood vessel; SB, subchondral bone; UFC, unmineralized fibrocartilage; TM, tidemark.

This study established sclerostin as an excellent functional marker for mature fibrochondrocytes in mineralized fibrocartilage. The progression of rapid mineralization in unmineralized fibrocartilage is orchestrated by ALP^+^ cells at the mineralization front, culminating in the ultimate expansion of unmineralized fibrocartilage after a decline in ALP^+^ cells (Figure 8A). In the epiphysis, the formation of fibrocartilage intricately correlates with bone replacement of hyaline cartilage. Mechanical forces transmitted through tendons, generated by muscle contractions, prove essential for the proper development of fibrocartilaginous entheses featuring mature sclerostin^+^ fibrochondrocytes (Figure 8B). A loss-of-function study highlighted sclerostin’s role in modulating the degree of mineralization and the stiffness profile of the fibrocartilaginous enthesis, crucial for maintaining mechanical tissue integrity (Figures 8C,D).

Both fibrocartilage and hyaline cartilage arise as avascular tissues (Benjamin and Evans, 1990; Shukunami et al., 2008; Takimoto et al., 2009). However, while hyaline cartilage involved in endochondral bone formation is transient and ultimately replaced by bone through vascular invasion (Hall, 2015), mineralized fibrocartilage in the enthesis undergoes rapid mineralization without significant cellular hypertrophy. Unlike epiphyseal mineralized hyaline cartilage, mineralized fibrocartilage persists into adulthood due to limited resorption of the mineralized fibrocartilage via osteoclasts from the adjacent epiphyseal bone marrow (Dyment et al., 2015). Immunostaining revealed that mature mineralized fibrocartilage expressing sclerostin lacks CD31^+^ blood vessels, underscoring its resistance to vascular invasion—a crucial characteristic subjected to mechanical loading from skeletal muscle through the tendon, although the underlying molecular mechanisms remain to be elucidated.

Postnatally, the development of the primordial hyaline cartilaginous enthesis evolves into a fibrocartilaginous enthesis in a well-coordinated manner, marked by changes in cell populations. A recent single-cell RNA-seq study (Fang et al., 2022) delineated distinct enthesis cell populations in the shoulder rotator cuff at P11, P18, and P56. As demonstrated in this study, postnatal enthesis formation correlates closely with endochondral bone formation in the epiphyses. Hypertrophic chondrocytes within the epiphyseal calcified hyaline cartilage, eventually replaced by bone, express various growth/differentiation factors (Kronenberg, 2003) that likely impact fibrocartilage formation in a paracrine manner. Notably, Indian hedgehogs secreted from prehypertrophic/hypertrophic chondrocytes in epiphyseal hyaline cartilage induce Gli^+^ cells, crucial for fibrocartilage mineralization (Schwartz et al., 2015). In *Scx*-deficient mice, with compromised tendons and delayed maturation of epiphyseal hyaline cartilage, enthesis formation is severely impaired, likely owing to a combination of reduced mechanical loading and diminished Ihh expression.

This study adopted Kawamoto’s method, allowing the tracking of enthesis formation in non-decalcified sections that closely mimic *in vivo* conditions. Fibrocartilaginous enthesis revealed the coexistence of mineralized hyaline cartilage and fibrocartilage. Among the mineralizing chondrocytes in these akin yet distinct cartilaginous tissues, epiphyseal hypertrophic chondrocytes exhibited Col2^+^/Col10^+++^/Col1^–^, while mineralized fibrochondrocytes displayed Col2^++^/Col10^+^/Col1^++^. Intriguingly, the fibrochondrocytes, expressing high levels of ALP and undergoing rapid mineralization, were significantly smaller than hypertrophic chondrocytes in epiphyseal hyaline cartilage. Notably, cellular hypertrophy and Col10 expression did not directly correlate with mineralization during enthesis formation.

The regulation of fibrocartilage width between the tendon and subchondral bone remains uncertain, but mechanical loading emerges as a pivotal factor. Each enthesis experiences unique mechanical loading based on its anatomical location, resulting in diverse enthesis structures and sizes (Benjamin and Ralphs, 1998; Benjamin et al., 2004). In the supraspinatus tendon enthesis of *Prx1Cre* ^*+*^ *;Scx* ^*flox/–*^ mice, impaired enthesis maturation was evident, with no discernible tidemark between unmineralized and mineralized fibrocartilage (Killian and Thomopoulos, 2016). Similarly, the Achilles tendon enthesis of *Scx* ^*Δ11/Δ11*^ mice exhibited defective fibrocartilage mineralization and underdeveloped epiphyseal hyaline cartilage and subsequent bone formation due to impaired mechanical loading, emphasizing its crucial role in the proper development of these tissues.

Mineral apposition in the fibrocartilaginous enthesis and subchondral bone occurs in opposing directions at the interface between the supraspinatus tendon and the bone, consistent with previous studies (Dyment et al., 2015). In hyaline cartilage, ALP activity starts low in proliferating chondrocytes but increases with cellular hypertrophy and extracellular matrix mineralization at primary and secondary ossification centers, as well as the growth plate (Cooper et al., 2013; Hallett et al., 2019). Our findings also demonstrate that ALP^+^ cells guide mineralized fibrocartilage formation at the mineralization front, extending towards the tendon midsubstance. In the fibrocartilaginous enthesis, a distinctive cell population expressing Gli1, a crucial mediator of Hedgehog signaling, contributes to postnatal development and regeneration of mineralized fibrocartilage (Schwartz et al., 2015; Schwartz et al., 2017). Gli1^+^/ALP^+^ cells may serve as progenitors for the formation of mineralized fibrocartilage. A parallel phenomenon is observed in secondary cartilages, like the mandibular condylar fibrocartilage, where ALP^+^ progenitors rapidly differentiate into hypertrophic chondrocytes, facilitating swift fibrocartilage mineralization (Shibata et al., 2006).

The mineral gradient is crucial for mitigating stress concentrations and dispersing mechanical loads at the tendon–bone interface (Schwartz et al., 2012; Tits and Ruffoni, 2021). Our investigation revealed that the expansion of mineralized fibrocartilage persisted until the decline in the number of ALP^+^ cells at the mineralization front. In chondrogenic ATDC5 cells, *Sost* knockdown via lentiviruses heightened mineralization (Yamaguchi et al., 2018). *Sost* deficiency led to enhanced mineralization, along with sustained ALP expression in fibrocartilage and bone, resulting in increased stiffness of both unmineralized and mineralized fibrocartilage, as assessed through AFM analysis. Interestingly, it has been reported that Gli1-expressing area was expanded during limb development in *Sost*-deficient mice (Collette et al., 2013). The hedgehog responsive Gli1^+^ progenitors give rise to fibrochondrocytes which mature to become mineralized fibrochondrocytes (Fang et al., 2022). Gli1^+^ cells may increase in the enthesis of *Sost*-deficient mice, resulting in enhanced mineralization. However, unlike Hyp mice with enthesopathy, a murine counterpart of human X-linked hypophosphatemia (Liu et al., 2018), the enthesis did not exhibit expansion in *Sost*-deficient mice compared to wild-type mice. Thus, sclerostin in fibrocartilage fine-tunes the degree of mineralization and the stiffness profile, maintaining the mechanical tissue integrity of the enthesis without significantly altering its morphology.

In this study, we demonstrated that fibrochondrocytes were Wnt-responsible by tracing Axin2 lineage cells in the enthesis. Sclerostin binds to LRP5/6, counteracting canonical Wnt signaling (Li et al., 2005; Yamaguchi et al., 2018), thereby fostering chondrocyte hypertrophy and subsequent extracellular matrix mineralization. Additionally, sclerostin functions as a BMP antagonist, inhibiting BMP-6-induced ALP activity in C3H10T1/2 cells (Winkler et al., 2003). Activation of these signaling pathways has been observed in the calcaneus of *Sost*-deficient mice (Krause et al., 2010). In fibrocartilage, sclerostin likely negatively regulates mineral deposition by controlling ALP activity through the suppression of Wnt and/or BMP signaling. Sclerostin governs bone remodeling by inhibiting bone formation and promoting bone resorption (Baron and Kneissel, 2013). Given *Sost* expression in mature fibrochondrocytes adjacent to the subchondral bone, it may also serve as a paracrine factor participating in bone remodeling.

Entheses represent interfaces between the elastic tendon and rigid bone, featuring a stiffness mismatch of nearly two orders of magnitude, subject to substantial mechanical demands (Tits and Ruffoni, 2021). The mechanosensitive properties of the tendon-enthesis-bone unit are vital for maintaining mechanical tissue integrity. Scx, a functional marker of tendons and ligaments, responds to tensile forces (Takimoto et al., 2015). Sclerostin, as a mechanosensitive molecule (Lin et al., 2009), exhibits upregulation with mechanical unloading leading to bone loss in osteocytes (Lin et al., 2009), while mechanical loading downregulates its expression (Robling et al., 2008). Fibrocartilage adapts to compression and/or shear stress, with the deep part compressed by the superficial part (Benjamin et al., 2006). Fibrochondrocytes within a mineralized matrix likely sense mechanical forces to regulate sclerostin expression. Ongoing studies aim to unravel how the mechanical force transmitted through the tendon is absorbed and converted to fibrocartilaginous entheses.

## Data Availability

The original contributions presented in the study are included in the article/Supplementary Material, further inquiries can be directed to the corresponding author.
