# Supplementary material for: Sclerostin modulates mineralization degree and stiffness profile in the fibrocartilaginous enthesis for mechanical tissue integrity
Source: Front Cell Dev Biol. 2024 Jun 4;12:1360041. doi: 10.3389/fcell.2024.1360041 (PMC11183276; doi:10.3389/fcell.2024.1360041)
Supplement: Supplementary file 1 [file DataSheet1.docx]

Supplementary Material

# Supplementary Figures and Tables

## Supplementary Figures

A

**0 W**

**1 W**

**2 W**

**4 W**

**5 W**

**6 W**

**Tamoxifen food**

**5 days**

**3 W**

**P45**

**Tomato/DAPI**


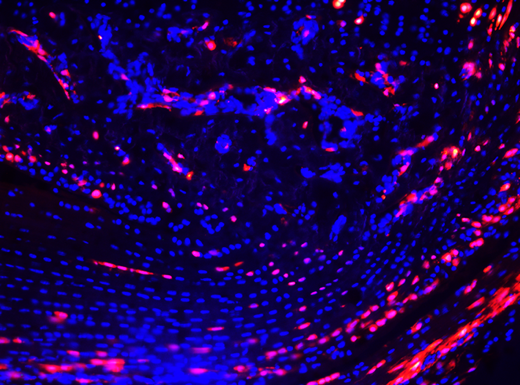


**FC**



**B**

**B**

**T**


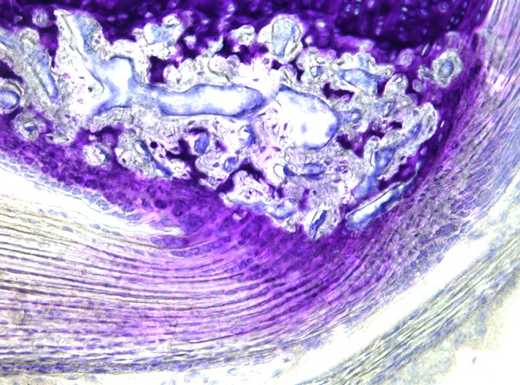

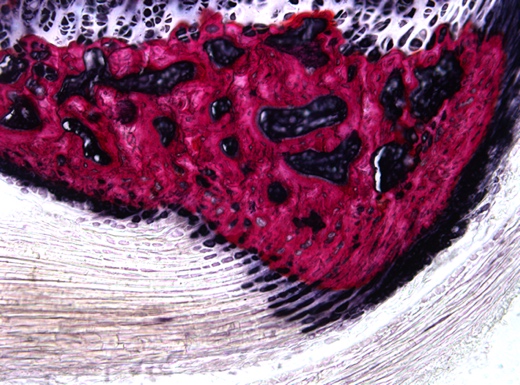


**C**

**D**

**TB**

**AR/ALP**

**FC**

**FC**

**B**

**T**

**B**

**T**

**Fig. S1.**

**Axin2 expressing cells in mature fibrochondrocytes of the Achilles tendon enthesis.**

Undecalcified frozen sections of the Achilles tendon enthesis were prepared from a 45-day-old *Axin2 -CreERT2;RosaTomato;2.3kb Col1* with the left first molar extraction socket after 5 days of tamoxifen diet **(A)**. Cre-mediated Tomato expression was detected as red fluorescence **(B)**. Sagittal sections were stained with TB **(C)** or AR/ALP **(D)**. The nuclei were stained with DAPI (blue). Data are representative of two age matched mice. Yellow arrows indicate fibrocartilage. Yellow dotted line in B indicates the boundary between fibrocartilage and subchondral bone. The Achilles and superficial digital flexor tendons are enclosed by white dotted lines in B. Abbreviations: T, tendon; FC, fibrocartilage; B, bone. Scale bars: 200 µm.

A


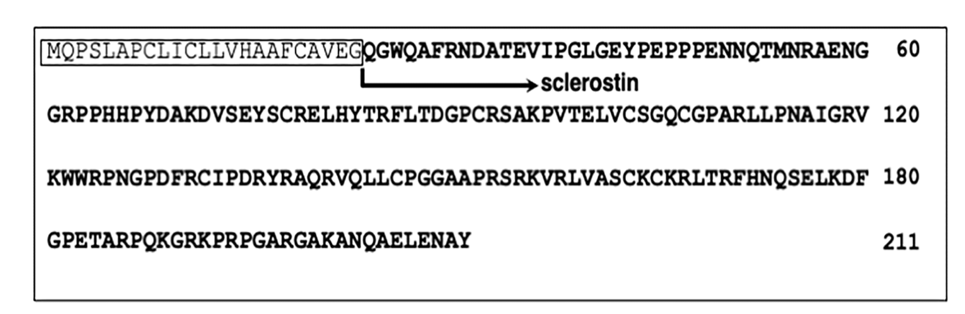

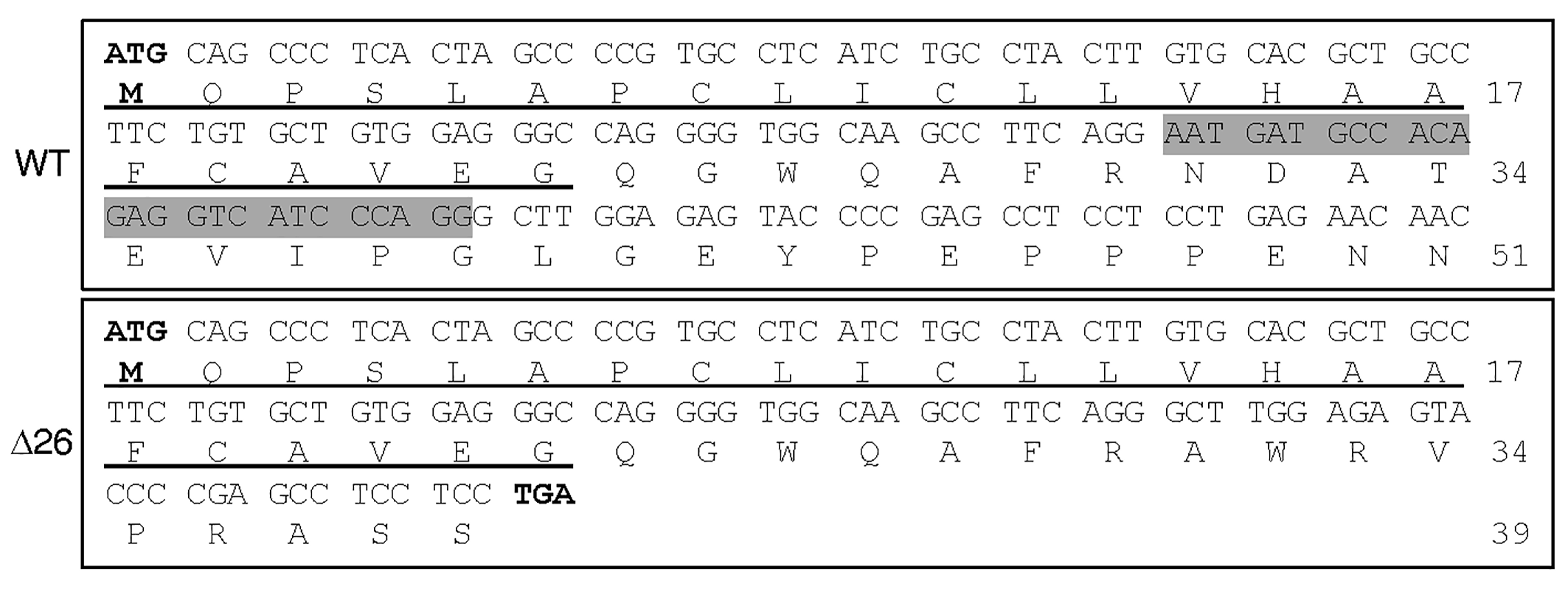


B

**C**


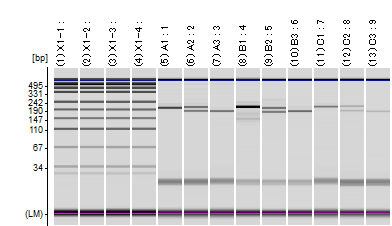


**Fig. S2.**

**Amino acid sequences of mouse sclerostin and frameshift mutations with early stop codon created by TALENs.**

(**A**) Mouse sclerostin comprises 211 amino acids, with the putative signal peptide enclosed. The sclerostin protein, shown in bold, contains 188 amino acids from Q24 to Y211. (**B**) Sequence of a mouse line with a 26-base pair deletion, with initiation and premature stop codons highlighted in black, respectively.(**C**) Gel images generated by MultiNA for genotyping PCR. The cropped images are enclosed with dotted lines (C) and further processed to present as Fig. 4B.


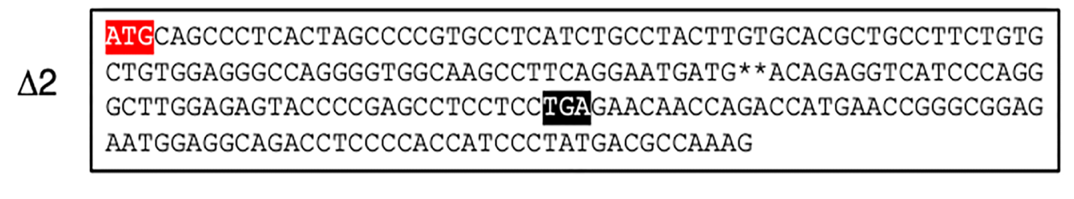

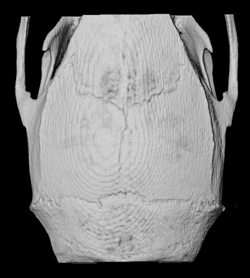

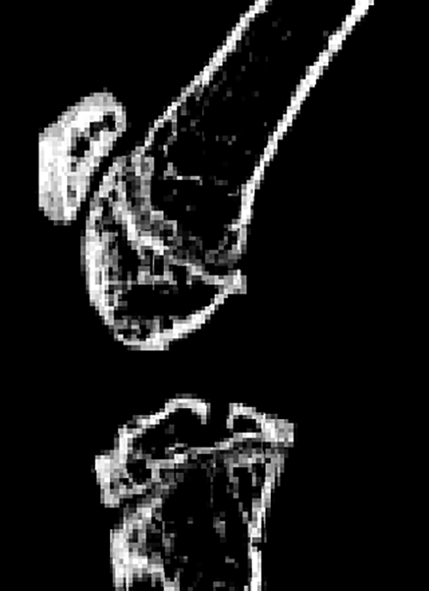

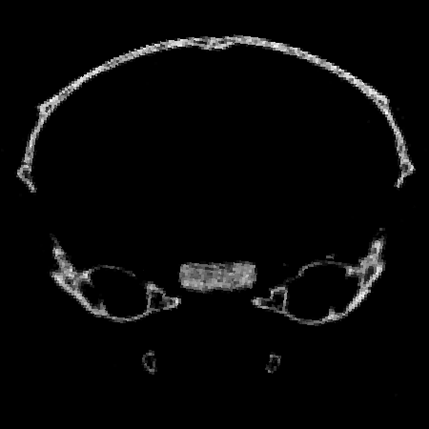

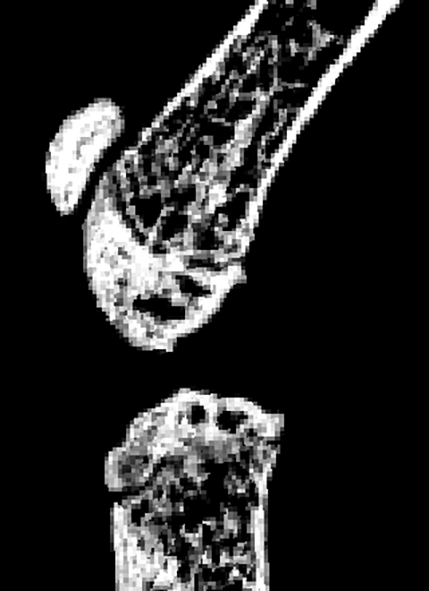

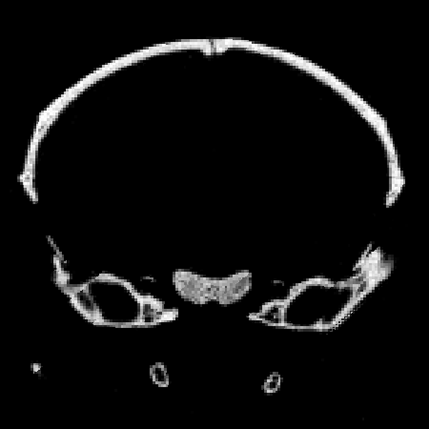

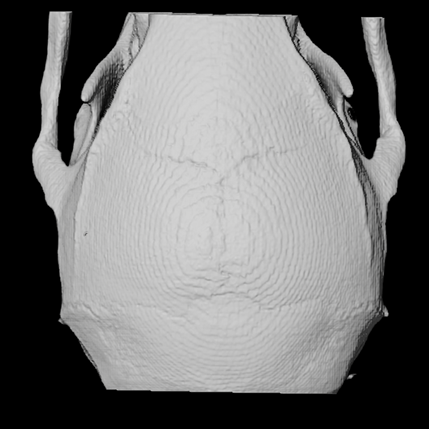


***Sost^Δ2/Δ2^***

***Sost^Δ2 /+^***

**fi**

**ti**

**pa**

**fi**

**ti**

**pa**


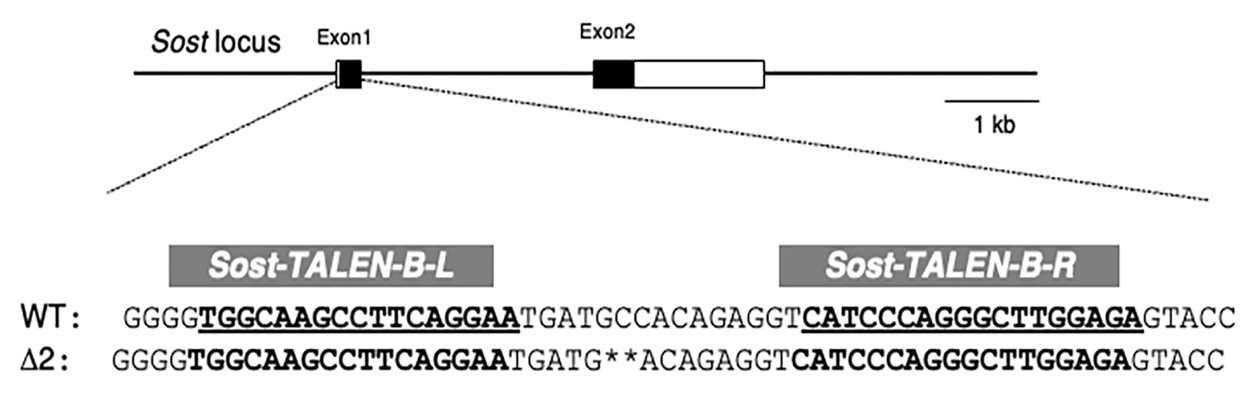


A

B

C

**Fig. S3. Increased bone mass in *Sost****^Δ^****^2/^****^Δ^****^2^* mice.**

**(A)** The genomic structure of *Sost* and TALEN target sequences in the mouse *Sost* locus. The left (L) and right (R) binding regions of *Sost-TALEN-B* are indicated by bold and underlined text. Sequences of wild-type (WT) and a founder mouse generated by microinjection of *Sost-TALEN-B-R/L* mRNAs. Nucleotide deletions are indicated by asterisks. (**B**) Sequence of a mouse line with a 2-base pair deletion, with the initiation codon and the premature stop codon highlighted in red and black. **(C)** Micro-CT images of knee joint and calvaria at P58 *Sost^Δ2/+^* mice and *Sost^Δ2/Δ2^* mice are shown. Abbreviations: pa, patella; ti, tibia; fi, fibula.

**250**

**150**

**100**

**75**

**50**

**37**

**25**

**10**


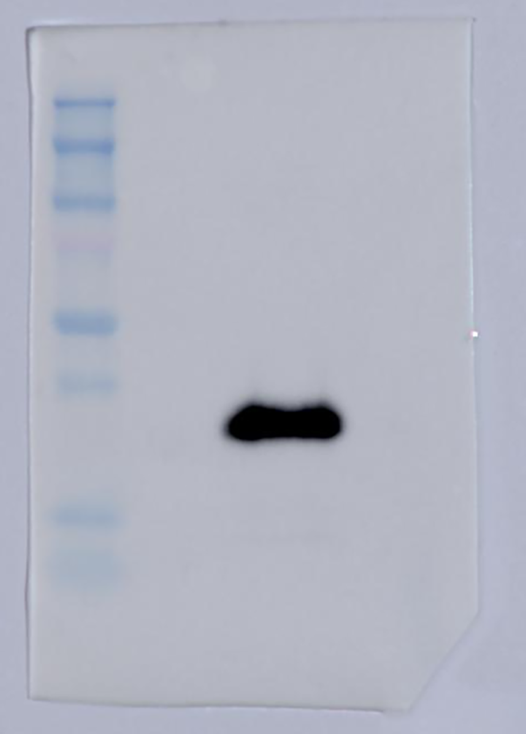


***Sost^+/+^***

***Sost^Δ26/Δ26^***

**kDa**

**Anti-Sclerostin**

**15**

**20**

**10**

**15**


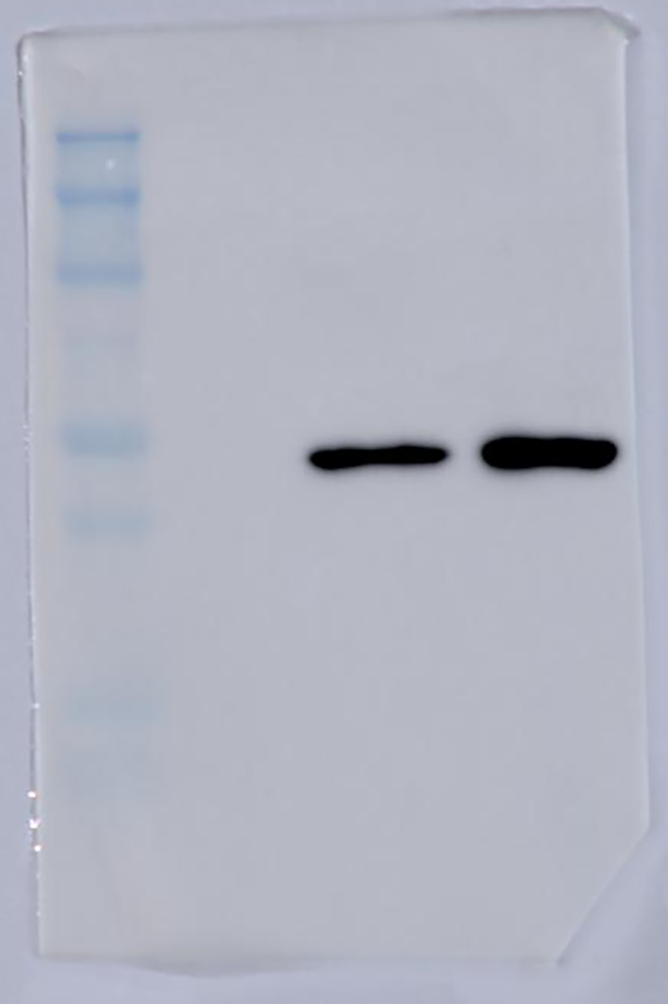


***Sost^+/+^***

***Sost^Δ26/Δ26^***

**kDa**

**Anti-GAPDH**

**250**

**150**

**100**

**75**

**50**

**37**

**25**

**20**

**Fig. S4.**

The full-length western blot images with molecular weight markers are shown. The cropped images are enclosed with red dotted lines and the processed image is presented as Fig. 4C.

**


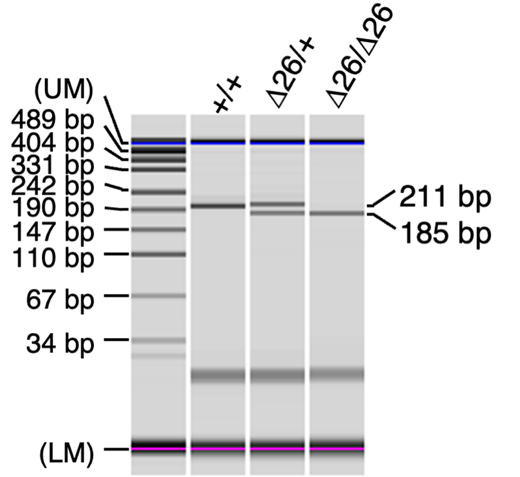
**
